# Ambulatory teaching: Do approaches to learning predict the site and preceptor characteristics valued by clerks and residents in the ambulatory setting?

**DOI:** 10.1186/1472-6920-5-35

**Published:** 2005-10-14

**Authors:** M Dianne Delva, Karen W Schultz, John R Kirby, Marshall Godwin

**Affiliations:** 1 Department of Family Medicine, Queen's University, Kingston, Ontario, Canada; 2 Faculty of Education, Queen's University, Kingston, Ontario, Canada

## Abstract

**Background:**

In a study to determine the site and preceptor characteristics most valued by clerks and residents in the ambulatory setting we wished to confirm whether these would support effective learning. The deep approach to learning is thought to be more effective for learning than surface approaches. In this study we determined how the approaches to learning of clerks and residents predicted the valued site and preceptor characteristics in the ambulatory setting.

**Methods:**

Postal survey of all medical residents and clerks in training in Ontario determining the site and preceptor characteristics most valued in the ambulatory setting. Participants also completed the Workplace Learning questionnaire that includes 3 approaches to learning scales and 3 workplace climate scales. Multiple regression analysis was used to predict the preferred site and preceptor characteristics as the dependent variables by the average scores of the approaches to learning and perception of workplace climate scales as the independent variables.

**Results:**

There were 1642 respondents, yielding a 47.3% response rate. Factor analysis revealed 7 preceptor characteristics and 6 site characteristics valued in the ambulatory setting. The *Deep *approach to learning scale predicted all of the learners' preferred preceptor characteristics (β = 0.076 to β = 0.234, p < .001). Valuing preceptor *Direction *was more strongly associated with the *Surface Rational *approach (β = .252, p < .001) and with the *Surface Disorganized *approach to learning (β = .154, p < 001) than with the *Deep *approach.

The *Deep *approach to learning scale predicted valued site characteristics of *Office Management, Patient Logistics, Objectives *and *Preceptor Interaction *(p < .001). The *Surface Rational *approach to learning predicted valuing *Learning Resources *and *Clinic Set-up *(β = .09, p = .001; β = .197, p < .001). The *Surface Disorganized *approach to learning weakly negatively predicted *Patient Logistics *(β = -.082, p = .003) and positively the *Learning Resources *(β = .088, p = .003).

Climate factors were not strongly predictive for any studied characteristics. *Role Modeling *and *Patient Logistics *were predicted by *Supportive Receptive *climate (β = .135, p < .001, β = .118, p < .001).

**Conclusion:**

Most site and preceptor characteristics valued by clerks and residents were predicted by their *Deep *approach to learning scores. Some characteristics reflecting the need for good organization and clear direction are predicted by learners' scores on less effective approaches to learning.

## Background

Medical care is increasingly delivered in the ambulatory care setting. Since learning in context improves learning it is appropriate and necessary that medical training occurs in the future practice setting [1,2]. Teachers must organize the setting and approach teaching in this context in order to maximize learning.

We developed a survey instrument to assess the site and preceptor characteristics that clerks and residents believe to be most valuable to their learning in the ambulatory setting using validated questionnaires and study group consensus [3]. Many items such as giving feedback and discussing clinical reasoning were valued by most learners, although there were some differences for learners from different specialties or at different training levels. A few site and preceptor characteristics, such as teaching in the patient's presence, thought to be important for learning were not valued. Learner preferences may be appropriate guides for teaching, but it is not clear that these preferences always support effective learning. Studies in higher education suggest that the approach to learning affects learning outcomes. The conceptual models of approaches to learning include both motivation for learning and the strategies to fulfill the motivation and are influenced by the context for learning. It is widely accepted that the deep approach to learning which includes an integrative approach to understanding, leads to improved learning outcomes while surface (or reproducing) approaches which depend on rote memorization, are less effective [4]. Learners adopting surface approaches to learning may prefer site and preceptor characteristics that support their surface learning; instructors who follow those preferences may inadvertently undermine their own teaching!

Bowen and Irby, reviewed the quality and costs of education in the ambulatory setting and described what is known and the gaps in our knowledge [5]. Many studies have been conducted in single institutions and in the traditional ambulatory specialties. Bowen and Irby suggest that a conceptual model examining the teacher-learner-setting framework is needed to understand the effectiveness of the ambulatory setting for learning.

Challenges in examining the effectiveness of a learning environment include the reliability of the evaluation method, confounding factors and the ability to generalize beyond single groups or institutions. One way to examine the complex interaction of the environment and the approach taken by learners in that environment is by measuring the approaches to learning in the workplace [6].

**Figure 1:**
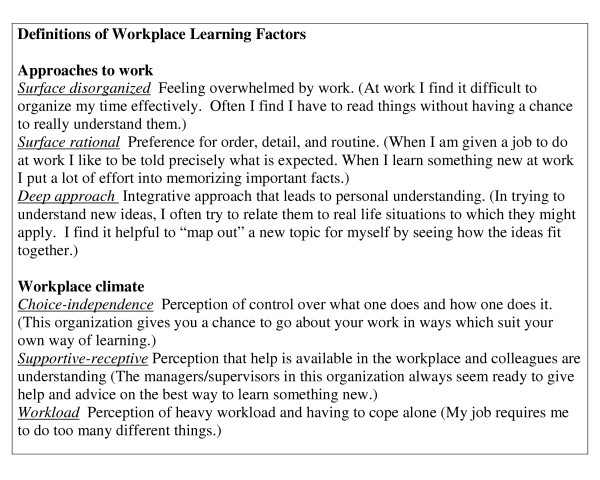
Definitions of Workplace Learning Factors (sample items).

The approach to learning, course perceptions and personal factors are known to interact to affect undergraduate learning [7-11]. Course demands influence university students to adopt surface, deep, or achieving approaches to learning. A deep approach to learning is motivated by an intrinsic desire for learning and involves strategies to form an integrated and personal understanding. In contrast, the surface approach to learning is motivated by fear of failure and is associated with rote memorization and a perception of heavy workload demands in the course. In studies of practicing physicians and clinical trainees (clerks and residents) we confirmed that perception of the workplace climate is associated with the approach to learning [12,13]. Perceptions of heavy workload are associated with surface disorganized approaches to learning (figure 1). A deep approach to learning is associated with perceptions of choice and independence and a supportive, receptive workplace climate (figure 1).

Our assumption in this study was that a deep approach to learning could be viewed as a surrogate for more effective learning and that a surface disorganized approach was less effective in most circumstances. Students who adopt a deep approach to learning may value characteristics of the learning environment and teaching that support this approach which differ from students who adopt less effective approaches to learning. It would seem appropriate then for teachers to attend to those site and preceptor characteristics that support a deep approach to learning. The purpose of this study was to answer the question: Do approaches to learning and perception of the workplace climate predict the site and preceptor characteristics valued by clerkship students and residents in the ambulatory setting?

## Methods

All medical clerks (n = 532) and residents (n = 2939) at the five medical schools in Ontario were invited to respond to a survey regarding the site and preceptor characteristics most valued in the ambulatory setting and to complete the Workplace Learning Questionnaire. The study was approved by the Queen's University Research Ethics Board.

Students rated 24 site characteristics and 38 preceptor behaviours on a Likert scale from 1 (very important for learning) to 5 (not at all important for learning) or D (detrimental for learning). (sample items in Table 1) The Workplace Learning Questionnaire developed by Kirby and colleagues was modified to include reference to preceptors as well as supervisors [14] The questionnaire includes 30 items pertaining to approach to learning and 15 items pertaining to perception of workplace climate (Figure 1). Responses are made on a 5 point Likert scale, ranging from 1(agree strongly) to 5 (disagree strongly).

**Table 1:** Definitions of site and preceptor factors (sample items rated from most important to least important)

**Precptor Factor**	**Definition**
Professional Role Modeling	Models professional behviours with staff and patients (Demonstrates effective interactions with support staff)
Teaching	Quality and efforts to provide good teaching (Discusses clinical topics in an organized way)
Learning Climate	Open and caring towards students and patients (Makes student feel like a valued member of the practice)
Feedback	The provision of timely and constructive feedback. (Gives constructive feedback)
Direction	Provides specific instruction on the student's role and is focused (Outlines specific task(s) to be done during a clinical encounter)
Patient Presence	Teaching with the patient present (Reviews case in the patient's presence)
Health Care System Interaction	Preceptor teaches about resource use. (Teaches use of community resources)
**Site Characteristic Factor**	
Office Management	Teaching skills related to the running of a practice (Teaching of time management skills)
Patient Logistics	Opportunity to see a number and variety of patients (Opportunity to see an adequate number of patients)
Objectives	Defines and meets objectives (Clearly defined site objectives for the rotation)
Learning Resources	Availability of resources in the clinic (Library resources available in the clinic)
Clinic Set-up	Proximity and educational orientation of the clinic (Close proximity of clinic to campus)
Preceptor Interaction	Effective teachers who are available and willing to demonstrate (Preceptors readily available)

Questionnaires were mailed in bulk to the undergraduate and postgraduate medical schools and forwarded to all clerks and residents with addressed and stamped return envelopes. Responses were anonymous and a separate card was returned for entry into a draw for a personal digital assistant or equivalent monetary prize. An email reminder was sent four weeks later and follow up mailings were sent 8 and 20 weeks after the initial mailing.

Multiple regression analysis was used to predict the preferred site and preceptor characteristics as the dependent variables by the average scores of the approaches to learning and perception of workplace climate scales as the independent variables. Hierarchical modeling did not alter the results.

## Results

Of the population surveyed, 1642 responded (47.3%). Participants with 10% or more missing data (those who did not respond to items (54) and those who responded "not applicable" (27)) were excluded, leaving 1561 respondents for analysis. Missing values for the remaining participants were replaced with item means. The distribution of respondents was compared to the distribution of clerks and residents in Ontario. The proportions of women, junior residents, family medicine residents and McMaster trainees responding were higher and Toronto trainees lower than the population surveyed [3,13].

Factor analysis revealed 7 preceptor factors and 6 site factors valued in the ambulatory setting [3]. These are defined in Table 1 with sample scale items. The factor structure and reliability of the Workplace Learning questionnaire were confirmed for the population [14]. Scales are presented in italics in the following discussion to emphasize that the associations are between scale scores.

Higher scores on the *Deep *approach to learning scale were associated with placing higher value on all of the preceptor and site characteristics valued by clerks and residents (table 2). Preceptor *Direction *was more strongly predicted by *Surface Rational *and *Surface Disorganized *scales than by the *Deep *scale (table 2).

**Table 2:** Prediction of valued preceptor characteristics by approaches to learning and perception of workplace climate. (β coefficients)

	Professional Role Modelling	Teaching	Learning Climate	Feedback	Direction	Patient Presence	Health Care System Interaction
Deep	.234**	.224**	.207**	.155**	.076*	.091**	.180**
Surface Rational	.077*	.179**	.101**	.111**	.252**	.118**	.104**
Surface Disorganized	.064	.013	.035	.015	.154**	.072	.048
							
Choice/Independence	-.046	-.056	.016	-.095*	.023	.066	.016
Workload	.094**	.043	.088*	.010	.070*	.012	.043
Supportive Receptive	.135**	.054	.065	.025	-.008	-.042	.058
Adjusted R^2^	.094	.092	.079	.036	.132	.036	.061

*p < .01, **p < .001

The *Deep *scale predicted valued site characteristics of *Office Management, Patient Logistics, Objectives *and *Preceptor Interaction *(table 3). The *Surface Rational *scale predicted valuing *Learning Resources *and *Clinic Set-up*. The *Surface Disorganized *scale weakly negatively predicted *Patient Logistics *and positively *Learning Resources.*

**Table 3:** Prediction of valued site characteristics by approaches to learning and perception of workplace climate. (β coefficients)

	Office Management	Patient logistics	Objectives	Learning Resources	Clinic Setup	Preceptor Interaction
Deep	.113**	.119**	.161**	.068	.078*	.086**
Surface Rational	.128**	.080*	.190**	.090**	.197**	.087**
Surface Disorganized	.031	-.082*	-.004	.088*	.070	.010
						
Choice/Independence	.047	-.017	.003	.020	.076	-.064
Workload	.071	.056	.074*	.051	.081**	.068
Supportive Receptive	.065	.118**	-.010	.064	.023	.050
Adjusted R^2^	.054	.044	.076	.033	.083	.021

*p < .01, **p < .001

Climate factors were not strongly predictive (tables 2 and 3). *Role Modeling *and *Patient Logistics *were predicted by *Supportive Receptive *climate. Perception of heavy workload (*Workload*) predicted valuing *Direction, Clinic Set-up *and *Objectives*. Perception of *Choice/Independence *in the workplace weakly and negatively predicted valuing *Feedback*.

## Discussion

In this analysis of a multi-site, multi-level and multi-specialty study we found that higher scores on the *Deep *approach to learning scale predicted all of the learners' preferred preceptor characteristics, supporting the implementation of these in the ambulatory setting. The *Deep *approach to learning was in general more strongly associated with the valued preceptor factors than the valued site characteristics except for *Direction*.

Giving direction was more valued by learners with higher scores on the *Surface Rational *and *Surface Disorganized *scales. One interpretation may be the need for these learners to know what is expected of them. Although struggling learners may require this support, too close direction may interfere with the development of independence and life-long learning skills. We have found that physicians who take a predominantly deep approach to learning are self-motivated for learning and prefer independent methods for continuing medical education [12]. In contrast, the physicians who score highly on surface approaches to learning are motivated by extrinsic factors such as regulating authorities or fear of a lawsuit [12]. As Samuel Johnson wrote, "*when a man knows he is to be hanged in a fortnight, it concentrates his mind wonderfully*" but it is likely a costly and ineffective approach for independent life-long learning. Relying too heavily on "what is on the exam" or what the preceptor wants does not allow for self-assessment and may lead to less effective outcomes.

Learners' approaches to learning are not one or another, but more or less of the approaches measured and will vary with the context. In this study we did not find a significant influence in the models of the perception of the workplace climate on valued site or preceptor characteristics. We did confirm earlier finding that the approach to learning and perception of the workplace climate are associated [6,12,13]. Thus, it may be that when the learning climate is stressful, learners may shift from a preferred approach to learning, to one that helps them survive. The surface rational approach may be necessary in a busy clinic when there is little time for reflection. In this setting, the organization or clinic characteristics may become more salient to the learner.

McManus has shown that approaches to learning are predicted by personality and learning styles [15]. Personality traits tend to be stable but learning styles and approaches to learning can be modified by formal education [16-18]. It may be important to encourage the struggling or dependent learner to become more self-directed. As workload is associated with surface approaches to learning, the challenge in the ambulatory setting will be to pace the patient volume and variety of problems to provide adequate time for learning with the aim of supporting deeper approaches to learning.

Dolmans and colleagues used path analysis to determine the importance of some variables influencing the effectiveness of student rotations at out-patient clinics in one medical school [19]. Input variables, such as organizational quality, number of students contemporaneously involved and available space, and process variables, such as patient mix and supervision, were analyzed in relation to one output variable: the students' evaluation of the effectiveness of the rotation. Supervision was the most important influence on the student's perception of the effectiveness of the rotation. This finding is consistent with the stronger prediction of valued preceptor characteristics than site characteristics in our study.

Dolman's model focused on organizational variables and did not take into account differences in the students and how they learn. Biggs' model suggests that the student's approaches to learning are input variables that interact with the organizational variables to affect what students do to learn [8]. The trend for site characteristics to be more strongly predicted by the *Surface Rational *approach to learning reflects the desire for good organization by some learners. Our findings extend Dolman's model to help teachers understand the different values placed on site and preceptor characteristics in the ambulatory setting by learners employing different approaches to learning.

This study has several limitations. First, the predictive power of approach to learning on valued site characteristics is small, suggesting that other factors we have not measured, such as personality, may be important in determining characteristics valued by the learners [17]. Ability, expectations and prior knowledge may also be important. Secondly, approaches to learning are influenced by many factors and do not represent an outcome of the learning experience. Thirdly, deep learners may be effective learners in any setting. It is not know if adapting the setting or changing preceptor behaviours to those valued by these learners will be helpful for learners who take less effective approaches. Further work is required to assess whether changes in preceptor or site characteristics affect learning outcomes or shift the learner's approach to learning.

## Conclusion

Most site and preceptor characteristics valued by clerks and residents were predicted by their *Deep *approach to learning scores validating these preferences. Preceptor characteristics are likely more important for learning than site characteristics. Some characteristics reflecting the need for good organization and clear direction are predicted by learners' scores on less effective, surface approaches to learning. These findings provide insight on the needs of more vulnerable learners.

## Competing interests

The author(s) declare that they have no competing interests.

## Authors' contributions

**A**ll authors participated in the conceptual planning and design of the study. DD and JK provided the statistical analysis and data interpretation. DD prepared the manuscript and all authors participated in interpretation and manuscript revision.

## Pre-publication history

The pre-publication history for this paper can be accessed here:


